# Predictive Analysis of Inflammatory Biomarkers in Functioning in Subjects with Bipolar Disorder

**DOI:** 10.1192/j.eurpsy.2026.10709

**Published:** 2026-07-31

**Authors:** G. Cano-Escalera, I. Gómez Grávalos, M. Melero González, A. Flores, M. Ramirez, I. Zorrilla, A. Gonzalez-Pinto

**Affiliations:** BIORABA, Department of Psychiatry, Hospital Universitario de Alava, CIBERSAM, UPV/EHU, Vitoria, Spain

## Abstract

**Introduction:**

Bipolar Disorder is a chronic illness characterized by manic and depressive relapses throughout its course. The literature presents several theories that attempt to explain the factors involved, one of which is the existence of an inflammatory state that causes neuronal toxicity.

In this study, a predictive analysis is proposed to evaluate the association between functionality, measured by the FAST test, and various inflammatory biomarkers in patients with bipolar disorder.

**Objectives:**

the objective of identifying markers that significantly predict functionality in this population.

**Methods:**

The design is an observational study based on a cohort of 50 subjects with bipolar disorder. Functioning was assessed using the FAST test, a validated tool for studying the functioning of patients with mental disorders.

Following the study by Rosa et al., a score of 11 was used as the cutoff point to establish the categories of good and poor functioning.

The inflammatory variables analyzed were IL6, IL10, 15dPGJ2, TAS, GPx, GSH, GSSG, BDNF, TrkB, TrkB-FL, and TBARS.

The selection of statistically significant variables (p < 0.05) in this cohort of bipolar subjects was performed using multivariate logistic regression, predicting factors associated with bipolar disorder and inflammatory biomarkers. The model was adjusted for the subjects’ educational level and metabolic syndrome.

**Results:**

The results show an increase in IL6 (p = 0.046) and IL10 (p = 0.044), which is associated with a significant decrease in functioning. High GSSG values (p = 0.049) also increase the likelihood of poor functioning. Finally, high total GSH levels appear to be a protective factor, decreasing the likelihood of poor functioning (p = 0.050).

Regarding the model’s performance, it presented an AUC of 0.975, indicating excellent discriminatory power.

**Conclusions:**

There is a proinflammatory state in patients with Bipolar Disorders that generates an environment of oxidative stress. Increased levels of these biomarkers are associated with worse results in functional assessments, so a possible explanation could be the accumulated neurotoxic effect throughout the course of the disease.

Adjusted logistic regression shows that the inflammatory biomarkers IL-6, IL-10, GSSG, and total GSH are significant predictors of functionality in bipolar patients, even considering educational level and metabolic syndrome as potential confounding variables.

**Disclosure of Interest:**

None Declared

